# Supplementary material for: Identification of photoperiod-induced specific miRNAs in the adrenal glands of Sunite sheep (Ovis aries)
Source: Front Vet Sci. 2022 Jul 22;9:888207. doi: 10.3389/fvets.2022.888207 (PMC9354845; doi:10.3389/fvets.2022.888207)
Supplement: Supplementary file 1 [file Data_Sheet_1.ZIP › Supplementary file/miRDeep2 file.zip!/miRDeep2 file/Know_Structure/SPLP42A3/oar-mir-106a.pdf]

miRBase precursor	: oar-mir-106a
Total read count	: 30
oar-miR-106a read count	: 30
remaining reads	: 0

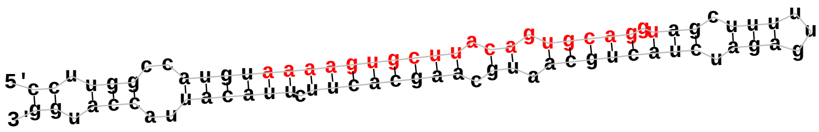

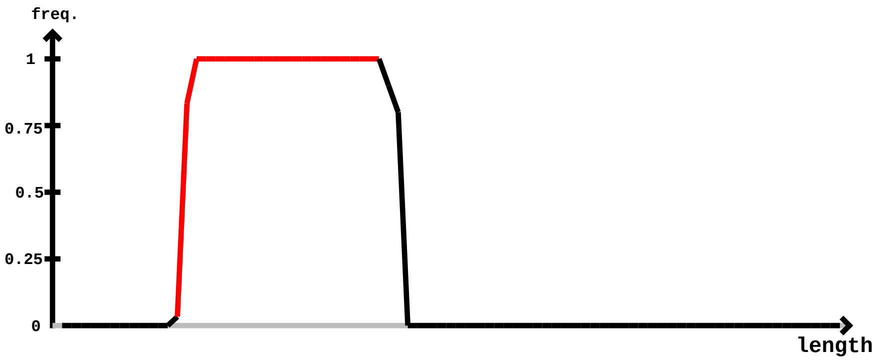


```
                oar-miR-106a
5'- ccuuggccauguaaaagugcuuacagugcaggauagcuuuuugagaucuacugcaaugcaagcacuucuuacauuaccaugg   -3'    exp
    ((.(((..((((((((((((((.((.(((((.(((.(((...))).)))))))).)).))))))))).))))))..))).))    reads       mm          sample
    ...........uaaaagugcuuacagugcaggu................................................    1           0           seq
    ............aaaagugcuuacagugcaggu................................................    2           0           seq
    ............aaaagugcuuacagugcaggua...............................................    2           0           seq
    ............aaaagugcuuacagugcagguag..............................................    20          0           seq
    .............aaagugcuuacagugcaggua...............................................    1           0           seq
    .............aaagugcuuacagugcagguag..............................................    3           0           seq
    .............aaagugcuuaUagugcagguag..............................................    1           1           seq
```
